# Toward High-Value Circular Pathways for Polymer Waste: Process–Structure–Property Strategies in Mechanical Recycling, Filament Re-Extrusion, and Additive Manufacturing

**DOI:** 10.3390/polym18050607

**Published:** 2026-02-28

**Authors:** Hanife Bukre Koc Gunessu, Gurcan Atakok, Menderes Kam

**Affiliations:** 1 Department of Mechanical Engineering, Institute of Pure and Applied Sciences, Marmara University, Kadikoy 34722, Istanbul, Türkiye; hbukre@gmail.com; 2 Department of Mechanical Engineering, Faculty of Technology, Marmara University, Maltepe 34854, Istanbul, Türkiye; 3 Department of Machine and Metal Technologies, Dr. Engin Pak Cumayeri Vocational School, Düzce University, Düzce 81700, Düzce, Türkiye; mendereskam@duzce.edu.tr

**Keywords:** circular polymers, mechanical recycling, fused deposition modeling, recycled PLA, filament re-extrusion, boron nitride, titanium, alumina, aluminum, process–structure–property

## Abstract

The global polymer waste burden has catalyzed a shift from linear “production–use–disposal” systems to circular models that prioritize recycling, reuse, and value retention. This article proposes an integrated, technology-ready roadmap for mechanical recycling and reuse of commodity and bio-based polymers via filament re-extrusion and Additive Manufacturing (AM). Building upon recent findings on performance envelopes of virgin vs. recycled Polylactic Acid (PLA) filaments processed by Fused Deposition Modeling (FDM), process parameter sensitivities (layer height, infill density) and their statistical optimization, and functional reinforcement routes (aluminum: Al, alumina: Al_2_O_3_, titanium: Ti, and Nano Boron Nitride: nano-BN), we articulate (1) a process–structure–property (PSP) mapping; (2) a low-defect, low-energy filament re-extrusion protocol; and (3) a graded-value strategy for upcycling mixed polymer streams. Across case analyses, we show that recycled PLA can achieve near-parity with virgin PLA when extrusion quality and printing parameters are controlled, and that ceramic/metal nanofillers enable thermal management and biocompatibility benefits crucial for durable reuse scenarios.

## 1. Introduction

Polymers’ versatility has enabled widespread deployment but also escalating waste. Circular polymer science aims to conserve value through recycling and reuse. Material-extrusion AM is strategically positioned to convert post-use polymer streams into customized high-value products with fine microstructural control via process parameters. Comparative studies of virgin vs. recycled PLA in FDM demonstrate feasibility of recycled filaments and emphasize the dominant influence of layer height and infill density on tensile, flexural, and impact strengths.

### 1.1. Introduction: From Linear to Circular Polymer Value Chains via FFF/AM

The confluence of mechanical recycling, filament re-extrusion, and fused filament fabrication (FFF) provides a practical route to close material loops for commodity and engineering polymers, with PLA often serving as a model system due to its bio-origin, biodegradability, broad commercial availability, and well-characterized performance envelope in AM [1,2,3,4,5,6]. While the AM literature historically tracked process innovation and prototyping efficiency, the present decade has seen a pivot toward high-value circularity, emphasizing the conversion of post-consumer and post-industrial polymer streams into reliable, application-ready feedstocks through process–structure–property strategies that maintain or even upgrade performance over iterative processing cycles [7,8,9,10,11,12,13]. This “upcycling” perspective requires the orchestration of (i) recycling physics (chain scission, thermo-oxidative history, crystallization and residual stress evolution), (ii) extrusion science (single vs. twin-screw, mixing, residence time control, drying and devolatilization, in-line monitoring), and (iii) AM process design (deposition thermal history, inter-raster cohesion, anisotropy control, infill architecture, and post-processing) [14,15,16,17,18].

PLA is a privileged testbed for circular AM due to its processability window, compatibility with diverse fillers, and expanding evidence base for both mechanical recycling and chemical (depolymerization) routes that widen end-of-life options [4,5,19,20,21,22]. The literature registers consistent sensitivity of recycled PLA (rPLA) to prints’ thermal–mechanical histories and to parameter-induced mesostructure (raster bonding, void content, mesoporosity, and spherulitic features), which together drive mechanical and dimensional outcomes and define a reproducible “operating window” for high-value applications [23,24,25,26]. The strategic question is how to transform heterogeneous polymer waste into high-performance AM feedstock while ensuring design-for-recycling, design-for-processability, and design-for-functionality are jointly met across use cycles [6,27,28,29,30,31].

To provide a clearer overview of the extensive research discussed in this introduction, the key literature, materials, and findings are systematically summarized in Table 1.

### 1.2. Process–Structure–Property (PSP) Framework for High-Value Circularity

A PSP view decomposes the circular AM system into unit operations and microstructure evolution pathways. In mechanical recycling, thermal reprocessing induces chain scission and viscosity decline, narrowing the processing window, altering crystallization kinetics, and potentially diminishing interlayer adhesion upon printing [11,13,32,33,50,51]. In re-extrusion, screw geometry, mixing intensity, temperature profiles, and die/haul-off conditions define diameter ovality, filler dispersion, and internal stress states, which then map into AM inter-raster bonding and anisotropy [14,15,16,52,53,54,55]. In printing, deposition temperature, build orientation, layer thickness, speed, and cooling govern interdiffusion across bead interfaces, void distribution, and orientation/crystallinity, ultimately controlling tensile, flexural, impact and wear properties [18,56,57,58,59,60,61]. Post-processing (annealing, heat treatment, and solvent smoothing) can re-equilibrate crystallinity and relax defects, sometimes compensating degradation incurred earlier in the cycle [24,44,45,62,63,64,65].

PLA’s semi-crystalline nature adds a tunable axis as follows: crystallization can be promoted by nucleating fillers (e.g., Al_2_O_3_, BN, TiN, and graphene nanoplatelets) and by thermal schedules, thereby improving heat deflection, creep resistance, and sometimes interlayer bonding if crystallization does not prematurely lock interfaces during deposition [34,35,36,37,38,66,67]. Conversely, excessive crystallization or poor interfacial compatibility can raise embrittlement risks or interfere with bead-to-bead coalescence; thus, interface engineering via compatibilizers, surface treatments, or binder phases often underpins robust structure development [21,41,42,68,69,70,71]. A circular PSP strategy therefore couples extruder-level dispersion and rheology control with printer-level thermal and geometric precision, and contextual post-processing and testing, all under DoE/Taguchi/RSM regimes for repeatable optimization [46,47,48,49,72,73,74,75].

Mechanical Recycling of PLA and Related Polymers: Degradation, Blends, and Property Retention: Repeated extrusion and printing cycles typically reduce PLA’s molecular weight via thermo-oxidative chain scission, weakening melt strength and altering crystal growth dynamics, yet multiple studies demonstrate maintainable mechanical performance through process optimization and additive strategies [13,32,33,76,77,78,79]. Early and mid-decade works compared virgin vs. recycled PLA prints and found that careful control of print temperatures, strand bonding, and infill can mitigate tensile losses, while annealing restores stiffness and reduces internal voids [24,32,33]. Use of recycled Polyethylene Terephthalate (rPET), polycarbonate (PC), Polypropylene (PP), and Polystyrene (PS) for filament (alone or in blends) underscores that distributed recycling can create durable AM feedstocks when drying [80,81,82,83,84,85,86], devolatilization, and screw/mixing parameters are tuned, and when filler systems or compatibilizers tailor interfacial mechanics [87,88,89,90,91,92].

**Figure 1:**
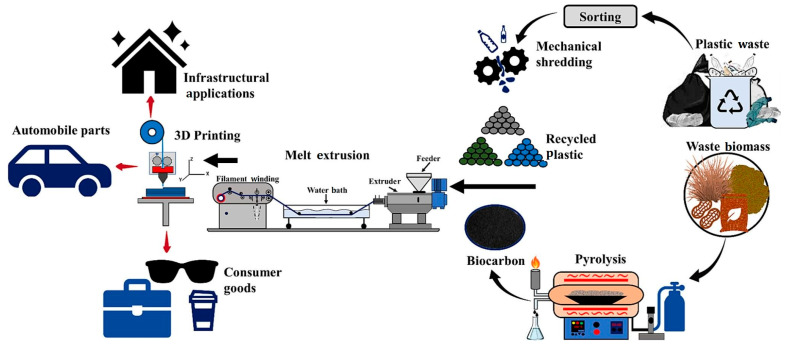
Schematic representation of the circular economy approach for PLA-based composites: integration of mechanically recycled plastics and waste biomass valorization through melt extrusion and FFF 3D printing applications. Reprinted from ref. [110].

As illustrated in Figure 1, the proposed circular economy framework for PLA-based composites integrates the following two primary streams: mechanically recycled plastic waste and waste biomass valorization [93,94,95,96,97,98]. The process begins with the collection and sorting of plastic waste, followed by mechanical shredding. Simultaneously, waste biomass undergoes pyrolysis to produce bio-carbon [99,100,101,102,103,104]. These components are then integrated through melt extrusion to develop sustainable filaments. Finally, these filaments are utilized in FFF 3D printing to manufacture high-value applications such as automotive parts, infrastructural components, and consumer goods, effectively closing the material loop [105,106,107,108,109].

Blends and bio-composites provide a second lever for circularity (Figure 1). PLA co-formulations (e.g., PLA/Polybutylene Adipate Terephthalate (PBAT) [111,112,113,114,115,116] or PLA with Polyethylene Glycol (PEG) plasticization) and natural fiber fillers (flax, kenaf, wood, nutshell, and olive pomace) can recover toughness [117,118,119,120,121] and damping while expanding end-of-life biodegradation options, provided fiber/matrix adhesion and moisture management are addressed [39,40,41,42,122]. For food packaging and film structures, heat-seal, adhesion to Ethylene Vinyl Alcohol (EVOH), and multilayer compatibilities have been documented, which informs post-consumer film recycling into functional blends for FFF—though controlling additive residues and barrier coatings remains challenging [123,124,125]. Chemical depolymerization/upcycling (e.g., to monomers or oligomers) is increasingly reported for PLA, offering escape routes from property attrition over many mechanical cycles and enabling rebuilding of molecular weight for new controlled-rheology feedstocks [19,21].

### 1.3. Filament Re-Extrusion: Equipment, Modeling, In-Line Monitoring and Control

**Figure 2:**
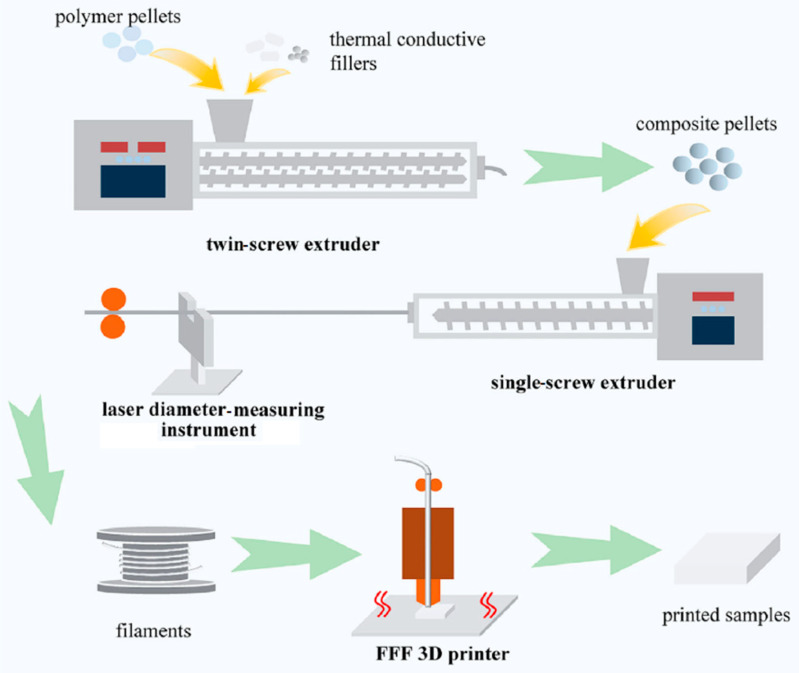
Schematic illustration of the composite filament fabrication process: compounding of PLA matrix with reinforcing fillers via twin-screw extrusion, followed by filament shaping and diameter calibration using a single-screw extruder with in-line laser metrology. Reprinted from ref. [137].

Extruder architecture (single- vs. twin-screw), screw design (mixing elements, kneading blocks), and process conditions (temperature profile, back pressure, and feed rate) govern dispersion of additives and recycled particulates, filament dimensional accuracy, and thermal history memory [14,15,16,17]. Twin-screw platforms enable stronger distributive and dispersive mixing for nanofillers (e.g., Al_2_O_3_, BN, graphene) and recyclate blends but raise shear-induced degradation risks without precise residence time and temperature management [15,17,126]. Studies on filament extruders for AM, including vertical extrusion prototypes and core-channel incorporation (solid/liquid additions), [127,128,129,130,131,132] show promising ways to introduce second phases or functional agents during re-extrusion with control over concentricity and ovality—critical for consistent feeding in printers [133,134,135,136] (Figure 2).

In-line metrology and control—notably diameter, ovality, melt temperature, and volumetric flow sensing—supports closed-loop regulation of puller speed and cooling profiles, reducing variability that would otherwise propagate to print quality and mechanical scatter [138,139,140]. The literature indicates that granule/pellet-fed AM (direct pellet extrusion) reduces reprocessing steps and energy overhead but demands robust material feeding and deposition stability mapping to avoid defects [141,142,143,144,145,146]; minimal-experiment frameworks have been proposed to delineate stable deposition envelopes as functions of temperature, speed, and flow [55,147,148,149]. Drying and dehumidification are essential to prevent hydrolytic degradation in polyesters like PLA, with mass/heat transfer modeling informing dryer design and cycle prescriptions, particularly for recycled inputs with unknown moisture histories [150,151].

### 1.4. Thermal Histories, Rheology, and Interlayer Cohesion in Printing

At the printer level, nozzle and bed temperatures, layer thickness, print speed, cooling airflow, and path planning determine an evolving thermal field that drives interdiffusion time across rasters and layers; adequate thermal contact enables healing of bead interfaces and reduces void content, improving tensile and fatigue performance [18,25,26,60]. The interaction of rheology (viscosity), crystallization kinetics, and cooling rate is nuanced for PLA as follows: too low viscosity impairs shape stability and dimensional accuracy; too rapid cooling undermines interlayer bonding, while too slow cooling risks slump or over-crystallization at interfaces [25,61,152]. Orientation effects are significant; build direction influences anisotropy in elastic and yield behavior and must be co-optimized with infill pattern/density to meet strength-stiffness targets [59,152,153].

Annealing (sub-Glass Transition Temperature: Tg/Melting Temperature: Tm) can reduce internal stresses, increase crystallinity, and boost stiffness and heat resistance of FFF-printed PLA, often improving tensile modulus and sometimes impacting resistance, though at the expense of dimensional changes that must be anticipated in design [24,44,45]. Chemical smoothing can reduce surface defects and act as a quasi-post-curing step for certain polymer systems, while also changing wettability and friction relevant to tribology [44,45]. For recycled PLA, careful annealing may recuperate part of the lost performance due to molecular weight drift by promoting more favorable spherulitic structures and mitigating porosity [24,32,79].

### 1.5. DoE, Taguchi, and RSM for Robust Optimization with Recycled Feedstocks

Given the multiple coupled parameters in re-extrusion and FFF, DoE methods (Taguchi, response surface methodology—RSM, and orthogonal arrays) have been widely adopted to identify interaction effects and derive robust settings for tensile, flexural, impact, and surface quality metrics [46,47,48,49]. Taguchi-based analyses on PLA/acrylonitrile butadiene styrene: ABS/Polyamide 12: PA12 show the relative contributions of layer thickness, raster angle, print temperature, and infill density to mechanical outcomes and production economics, guiding trade-offs for circular feedstocks where viscosity, moisture and contamination variability are higher [154,155,156]. Orthogonal experiments and RSM approaches are increasingly used to capture non-linear interactions, for instance, between nozzle temperature and print speed or infill density and raster orientation, which directly affect interlayer bonding strength and porosity distributions [49,157,158].

Recent studies [159,160,161,162,163,164] focused specifically on PLA with recycled content or tough PLA grades show that process window re-centering via Taguchi/RSM can restore or even exceed baseline virgin-PLA properties in tensile and Charpy impact, highlighting that on-machine optimization is a powerful lever to offset upstream degradation [165,166,167,168]. Moreover, vibration control of printer mechanics (gantry dynamics, acceleration profiles) has been tied to mechanical property scatter and surface roughness; mitigation strategies (e.g., damped bushings, tuned jerk/acceleration) can be integrated into DoE frameworks for consistent interlayer fusion with recycled filaments [169,170,171,172].

### 1.6. Filler Systems and Composite Design: Ceramic, Metallic, Carbonaceous, and Natural Fillers

Ceramic nanofillers such as alumina (Al_2_O_3_) and titanium nitride (TiN) demonstrably enhance tensile, flexural, impact, and thermal properties of PLA when well dispersed; Al_2_O_3_ also acts as a nucleating agent, improving dimensional stability and heat resistance [34,35,36,173]. Graphene nanoplatelets (GNPs) provide stiffness, strength, and sometimes improved dimensional accuracy at modest loadings, though balancing electrical percolation and printability is non-trivial [37,38,174]. Boron nitride (BN) and hexagonal BN (h-BN) contribute to thermal conductivity enhancements and wear resistance improvements, with multiple groups demonstrating strong property gains in 3D-printed composites [175,176,177]. For biomedical scaffolds, PLA/Ti and PLA/hydroxyapatite/titania systems pair mechanical reinforcement and osteoconductivity with FFF-compatible rheology, enabling porous architectures that support biomineralization and favorable cell responses [178,179,180].

Metal particle-filled PLA (e.g., Al, brass, and Ti) affects interlayer delamination propensity and surface finish [181,182,183,184,185]; process parameter optimization and heat treatments have been shown to temper embrittlement and improve cohesion [186,187,188]. The synergy of graphene + alumina in PLA has been specifically highlighted for thermal conductivity and mechanical property improvement, aligning with the PSP strategy of pairing nucleation sites and 2D reinforcement to tailor crystallinity and crack deflection pathways [37,189,190,191,192,193,194]. In parallel, natural fiber/natural filler composites (kenaf, flax, wood, nutshells, and olive pomace) demonstrate density reduction, stiffness improvements, and eco-profile advantages when fiber surfaces are properly treated and moisture is controlled during printing; these systems are a key bridge between biomass valorization and AM circularity [39,40,41,42,43].

Interface engineering remains a constant theme: without compatibilization or surface functionalization, nanofillers can agglomerate and act as crack initiators; similarly, fiber pull-out reduces energy absorption unless matrix–fiber adhesion is elevated by coupling agents or plasma/chemical treatments [21,68,195]. For recycled matrices, compatibilization can also mask molecular weight variability and help maintain viscosity in the printable window, improving bead coalescence and dimensional control [30,126]. Finally, the literature on continuous-fiber printing underscores that in-nozzle impregnation strategies allow higher load transfer, but waste stream reintegration is more complex; hybrid circular strategies may involve mechanical regrind of offcuts into short-fiber feedstock for subsequent re-extrusion and printing [196,197].

### 1.7. PLA as a Circular Platform: Biodegradability, Biocompatibility, and Advanced Applications

The corpus on PLA monomer synthesis, polymerization routes, molecular weight build-up, and application breadth establishes a robust platform for circular AM experimentation, including bio-medical, food packaging, and engineering components with tailored mechanical and barrier properties [4,5,11,125]. PLA’s biobased origin and biodegradation pathways (under industrial composting, and in certain laminated bio-composites) feed into the sustainability narrative, though context-appropriate end-of-life scenarios are essential to avoid performance vs. degradability trade-offs in durable goods [6,39,124]. Functional biomedical scaffolds fabricated via FFF demonstrate tunable pore size, wall thickness, and infill topology; PLA/Ti and PLA/hydroxyapatite frameworks show improved biocompatibility and mechanical proportioning to match bone tissue environments [154,178,179]. PLA composite films (including BN-modified films) for packaging point to thermal management and barrier enhancements while remaining compatible with thermoforming and potential re-extrusion routes [35,37,175,198,199,200].

Given PLA’s print-temperature sensitivity, healing window management during deposition (time above glass transition for interdiffusion) is vital, especially for recycled batches (e.g., rPLA) where viscosity and crystallization kinetics shift with prior history [25,26,61]. Dimensional accuracy and surface roughness can be tuned via infill design, motion precision, and post-treatments, supporting functional tolerances required for tooling, fixtures, and lightweight sandwich structures that exploit AM’s geometric freedom [61,201,202].

The literature also documents orientation-dependent elasticity and yield behavior, emphasizing that AM design rules must explicitly embed anisotropy considerations for recycled PLA to reach parity with injection-molded baselines [26,59,203,204,205].

### 1.8. Testing Standards and Metrology for Circular AM

Reliable comparison across studies requires standardized testing. The use of Organization for Standardization (ISO)527 for tensile testing and ISO 180 for Izod impact provides a common basis for mechanical benchmarking of rPLA and PLA composites, enabling meta-analyses and cross-study comparisons in optimization campaigns [206,207]. Works coupling mechanical tests with fractography, porosity quantification, and dimensional metrology (e.g., optical/Computed Tomography (CT) scans) help map process parameters to defect modes (voids, lack-of-fusion, and inter-bead cracks) and correlate them to strength reductions or premature failure [61,152]. In circular contexts, moisture content, melt flow index, and thermal analysis Differential Scanning Calorimetry: DSC/Thermogravimetric Analysis: TGA before and after recycling cycles are indispensable for diagnosing chain scission, crystallinity changes, and stabilizer loss, informing both extrusion settings and print recipes [11,126,208].

### 1.9. Post-Processing: Annealing, Heat Treatment, and Surface Conditioning

Annealing protocols for PLA prints (e.g., 70–120 °C for prescribed dwell times) increase crystallinity, reduce internal stresses, and may enhance modulus and heat deflection temperature; such treatments are effective for both virgin and recycled PLA, though dimensional shrinkage and warpage must be managed via fixtures or design over-build [24,44]. Heat treatments for metal-filled PLA (Al, Ti, and brass) have been studied, showing tensile strength benefits and reduced delamination when coupled with optimized extrusion/print temperatures, as metal particles modulate thermal gradients and interlayer cooling [186,187,188,209]. Chemical surface treatments and solvent polishing can smooth asperities, improve interfacial adhesion in bonded assemblies, and reduce stress concentrators—useful for recycled composites where surface defectivity may be higher [44,210,211].

### 1.10. Sustainability, Distributed Recycling, and Energy Considerations

Distributed recycling via fused particle fabrication (FPF) and recyclebots realizes community-scale circularity by converting waste flakes (PLA, PET, and PC) directly into feedstock, minimizing logistics and enabling localized, on-demand manufacturing [89,90,212]. The energy payback time for Photovoltaic (PV)-powered recyclebot systems has been modeled, supporting environmental viability of maker-space recycling ecosystems under realistic throughput and duty cycles [213,214]. Barrier analyses for maker communities highlight challenges in quality assurance (diameter control, contamination, and moisture) and user training, recommending in-line sensing, drying protocols, and materials labeling to improve part reliability and safety [215,216]. Broader sustainability assessments of FFF emphasize energy consumption, material efficiency, and emission control (ultrafine particles) as design targets for future machines and processes [217,218,219,220].

Studies comparing virgin vs. recycled AM parts indicate that properly optimized rPLA can deliver engineering-grade performance, particularly in non-critical structural roles or where hybrid reinforcement (e.g., nanoparticle nucleants, natural fibers) is acceptable [13,32,79]. The use of post-consumer recycled PET and polyolefin matrices with activated carbon or biocarbon underscores pathways to functional property restoration (stiffness, wear, and dielectric behavior), widening the set of “high-value” applications rather than relegating recycled polymers to low-demand use cases [91,221,222]. Policy and ecosystem levers—including Small- and Medium-sized Enterprise (SME) support, sector incentive reports, and education initiatives—frame the socio-technical infrastructure necessary for widespread adoption of circular AM [223,224,225] (KOSGEB. Kosgeb-e Journal (Small and Medium Enterprises Development Organization of Turkey), December 2020, Available online: kosgeb.gov.tr, accessed on 12 February 2026).

### 1.11. Application Case-Spaces: Tooling, Sandwich Structures, and Biomed

The AM literature identifies lightweight sandwich structures, honeycomb and graded foams, and tooling/fixtures as sweet-spots for circular PLA and composites, because infill architecture can compensate material property deficits by structural optimization [201,226,227,228]. Studies show that graded infill density and innovative internal architectures can significantly elevate specific stiffness and energy absorption, aligned with topology-optimized product design strategies [201,226,229]. For biomedical applications, PLA-based scaffolds and tympanic membrane patches demonstrate the feasibility of biofunctional geometries and the compatibility of PLA with cell-friendly environments; metal-/ceramic-reinforced scaffolds via FFF advance osteointegration while maintaining printability [154,178,179,230]. In dielectric and thermal management contexts, barium titanate-loaded ABS and graphene/alumina-modified PLA point to smart functional devices produced with material extrusion, blending circularity with device-level performance [37,231].

### 1.12. Parameter Sensitivities: Build Direction, Infill, Speed, and Temperature

**Figure 3:**
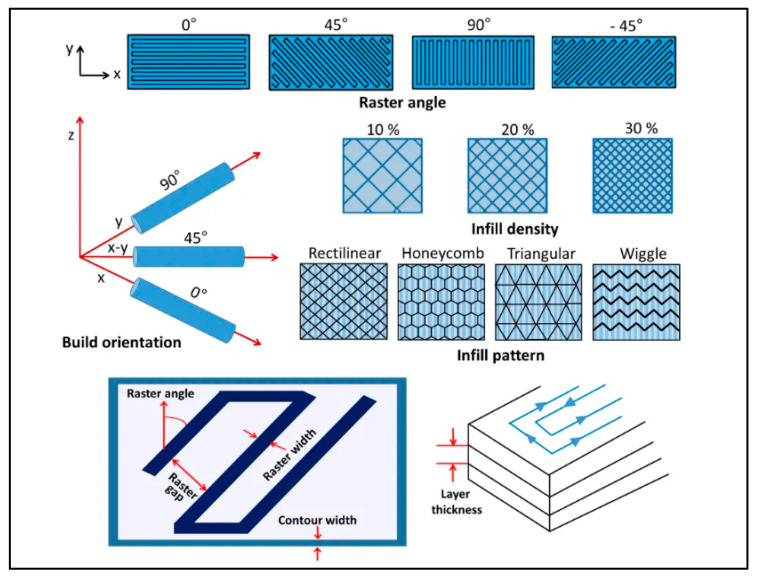
Schematic representation of critical FFF process parameters. Reprinted from refs. [233,234].

Build orientation is among the dominant factors for strength and toughness due to layer-wise anisotropy; aligning principal stress with bead paths can dramatically increase load-bearing capacity, particularly for rPLA where interlayer bonding margins may be narrower [59,152,232] (Figure 3).

Infill density and pattern (e.g., honeycomb, gyroid, and rectilinear) modulate energy absorption and failure modes; works demonstrate that infill strategies can offset material property variances from recycling, making them primary design handles for circular AM [165,201,235,236]. Nozzle temperature and speed control healing time and cooling rate; orthogonal experiments and RSM studies demonstrate non-linear optima, particularly for metal-/ceramic-filled PLA, where thermal conductivity of the filler accelerates cooling and may necessitate higher nozzle setpoints or reduced print speeds [47,186,237,238]. Dimensional accuracy is co-determined by plate–extruder motion precision, shrinkage on cooling, and over-extrusion/under-extrusion balance, all of which require calibration tailored to the rheology of recycled lots [61,239].

### 1.13. Case of PLA + Nanoceramics/Graphene: Synergistic Nucleation and Property Gains

The PLA + Al_2_O_3_ + graphene synergy exemplifies how hybrid nucleation and reinforcement co-improve mechanical and thermal properties: alumina nucleates crystallization and stiffens, graphene adds load transfer and bridges microcracks, jointly elevating thermal conductivity for heat management while preserving printability at moderate loadings [37,38,240]. TiN nanofillers deliver substantial mechanical reinforcement at low volume fractions, with thorough dispersion critical to avoid nozzle clogging and ensure uniform interlayer bonding [36]. BN/h-BN raises through-bead thermal conductivity, enabling faster heat dissipation; in AM, this can both help and hinder: it reduces thermal gradients (lower residual stress) but may shorten healing windows, making parameter re-centering necessary [175,176,177]. Collectively, these studies argue that filler selection in circular AM should consider not only target properties but also thermal transport impacts on printing physics, demanding co-optimization of extrusion and FFF [34,36,37].

### 1.14. Metal-Filled and Functionally Filled PLA: Post-Processing and Reliability

Aluminum- and brass-filled PLA filaments present dimensional and surface challenges, including increased abrasion on nozzles and rougher bead interfaces; nevertheless, parameter optimization (higher nozzle temperatures, controlled speeds) and post-heat treatments can recover tensile strength and improve delamination resistance [186,187,188]. Titanium-filled PLA scaffolds for biomed benefit from Ti’s biocompatibility; FFF of PLA/Ti composites with tuned pore architectures has yielded mechanically viable and bioactive constructs [154,178]. Comparative analyses against pure PLA prints confirm that interface and dispersion govern relative performance gains; powder morphology, surface chemistry, and loading fraction must be matched to extruder mixing and FFF conditions to avoid embrittlement [178,241,242]. For electrical/dielectric functions, barium titanate in ABS demonstrates tailorable dielectric constants, suggesting analogous circular routes for functional rPLA via hybrid fillers [231,243].

### 1.15. Natural-Fiber and Biomass-Derived Systems: Interface, Moisture, and Processing

Kenaf, flax, wood, nutshell, and olive pomace reinforcements in PLA leverage biomass streams for low-density, sustainable composites; fiber surface treatments (alkaline, silane) and compatibilizers improve adhesion, mitigating fiber pull-out and enabling higher toughness or specific stiffness [39,40,41,42]. However, moisture sensitivity of both PLA and natural fibers necessitates thorough drying and controlled storage to prevent hydrolysis during extrusion/printing; recycled streams exacerbate this sensitivity, demanding robust dryer design and real-time monitoring [126,150,151]. Printing parameters must be tuned to limit thermal degradation of biofillers and maintain dimensional integrity, while post-annealing can stabilize geometry and crystallinity, supporting applications in sandwich cores and bio-functional parts [43,201,244].

### 1.16. Reliability, Defects, and Treatments in Recycled Filaments

Recycled filaments exhibit greater lot-to-lot variability in MFI, moisture content, and contamination, leading to diameter fluctuation, stringing, under-/over-extrusion, and voiding if not effectively controlled [135,139,245]. Works on dimensional accuracy, surface roughness, and defect density establish process windows and correction strategies (e.g., calibrated extrusion multipliers, retraction tuning, and active chamber temperature control) that stabilize deposition [18,61,152,202]. Thermal annealing remains a convenient pathway to reduce internal defects; solvent/chemical treatments post-print can seal surface micro-porosity, beneficial for fluid-handling or coating adhesion needs [24,44,232]. In aggregate, in-line sensing, closed-loop control, and statistical monitoring (SPC) are essential to elevate recycled filaments to production-grade reliability [138,140,149].

### 1.17. Beyond PLA: ABS, PA12, PET, PC, and Multi-Polymer Systems

While PLA dominates circular AM studies, ABS, PA12, PET, and PC-recycled feedstocks are gaining traction, with performance maintained over multiple recycling cycles when process parameters are adapted to viscoelastic changes and filler strategies [89,246,247,248]. Recycled PET has been extruded into functional filaments and printed components; activated carbon/biocarbon fillers aid mechanical optimization, strengthening the case for post-consumer streams in engineering parts [91,221,222]. PA12 powder recovered from SLS has been repurposed for FFF, illustrating cross-process recycling loops between AM modalities [248,249]. These advances suggest a polymer-agnostic circularity template where material characterization, extruder configuration, and FFF parameterization are tailored to polymer class and recycling history [246,248,250].

### 1.18. Health, Safety, and Environmental Considerations

Material extrusion AM emits ultrafine particles and volatile species, with emission rates influenced by infill, temperature, and polymer class, motivating enclosure, ventilation, and filtration best practices—particularly important when unknown additives or contaminants ride along in recycled feedstocks [217,219]. Life-cycle analyses of FFF processes encourage energy-aware parameterization (e.g., minimizing reheating cycles, optimizing build orientation for fewer supports) and consolidation of parts to reduce assembly operations, aligning circularity with process sustainability [218,220]. The maker movement perspective adds behavioral and organizational dimensions—training, quality norms, and maintenance routines determine whether distributed recycling yields truly durable and safe parts [203,204,205,213,215,216].

### 1.19. Knowledge Gaps and Research Frontiers

Several frontiers remain for high-value circular pathways in AM, such as the following: in-line molecular weight and rheology sensing during re-extrusion to detect chain scission and optimize temperature/residence time on the fly, potentially combining Near-Infrared/Infrared (NIR/IR) spectroscopy with Melt Flow Index (MFI) proxies [138,139,140].

Closed-loop filament diameter and ovality control with predictive models that incorporate cooling line dynamics and puller speed disturbances, critical for recycled blends with variable thermal properties [138,149,245].

Interface-centric compatibilization tailored to recycled matrices: scalable surface treatments for nanoparticles and fibers that are robust to moisture and contaminant profiles [21,68,195].

Multi-objective optimization unifying mechanical, thermal, dimensional, and environmental targets, integrating DoE/Taguchi/RSM with digital twins of extrusion and printing [46,47,49,251].

Hybrid circular routes combining mechanical and chemical upcycling to rebuild molecular weight of degraded PLA and other polyesters while preserving color/additive functionality [19,21,183,184,185,252].

Standardized durability and fatigue protocols for recycled AM parts to validate long-term performance across climates and load spectra [26,206,249].

## 2. Protocols for Filament Re-Extrusion and Additive Manufacturing

Methodologies across source manuscripts to produce an implementable protocol spanning feedstock preparation (drying, segregation, and granulation), twin-screw compounding for composites (Al_2_O_3_ 1–4 wt%, BN 1–3 wt%, and Ti 1–10 vol%), single-screw filamentizing to 1.75 ± 0.05 mm with inline laser control, and FFF specimen fabrication per ISO 527/178/180 [206,207,253]. Statistical design follows Taguchi orthogonal arrays and Analysis of Variance (ANOVA); characterization includes Scanning Electron Microscopy (SEM), DSC/TGA, Raman/X-Ray Diffraction (Raman/XRD) and, where applicable, thermal conductivity measurements (laser flash/Hot Disk) are consolidated.

Design and overall approach: The experimental and review methodologies reported across three source documents to produce a single, implementable protocol suitable for both recycled PLA (Re-PLA) and PLA-based composites reinforced with Al, Al_2_O_3_, Ti and BN are harmonized. The workflow follows the following five stages: feedstock preparation, compounding/filamentizing, FFF specimen fabrication, property characterization, and statistical optimization.

**Figure 4:**
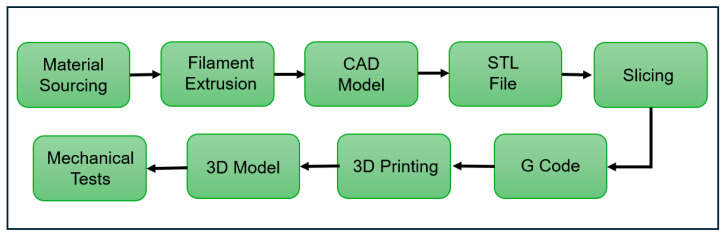
Flowchart of experimental methodology from raw material to mechanical testing. (Generated by the author).

Figure 4 outlines the specific experimental steps taken to apply this framework, covering the transition from material sourcing and filament extrusion to 3D printing and final mechanical characterization.

Feedstock preparation (Re-PLA and virgin PLA): Failed prints, support structures and end-of-life parts are segregated by polymer type and color, washed to remove oils and contaminants, and air-dried. Granulation to 3–5 mm pellets is followed by oven drying (60–65 °C, 4–6 h) to suppress hydrolysis, a known cause of molecular-weight loss and brittle failure in PLA. Moisture content is monitored using a simple mass-loss gravimetric check or a handheld moisture meter; specimens exceeding 0.5 wt% moisture are re-dried. For sustainability blends, Re-PLA:PLA ratios of 25:75, 50:50, 75:25 and 100:0 are prepared to evaluate the performance trade-offs and determine feasible circularity windows.

Compounding and filamentizing (composites and blends): Reinforcement and additive packages are prepared at low loadings to preserve printability as follows: alumina nanoparticles (Al_2_O_3_) at 1–4 wt% for hardness/wear, h-BN nanosheets at 1–3 wt% for thermal management, and titanium particles (or TiN ceramics) at 1–10 vol% for ductility/biocompatibility. Masterbatches are melt-mixed in a co-rotating twin-screw extruder with moderate shear (180–200 °C barrel profile) and neutral screw elements to minimize degradation, then pelletized. Filamentizing is performed on a single-screw line fitted with inline laser diameter control targeting 1.75 ± 0.05 mm; vacuum venting removes entrained bubbles, and a two-stage water bath (20–25 °C then 15–18 °C) stabilizes crystallization and surface finish. Optional in-line filters (100–200 μm) mitigate agglomerates that can cause nozzle clogging.

**Figure 5:**
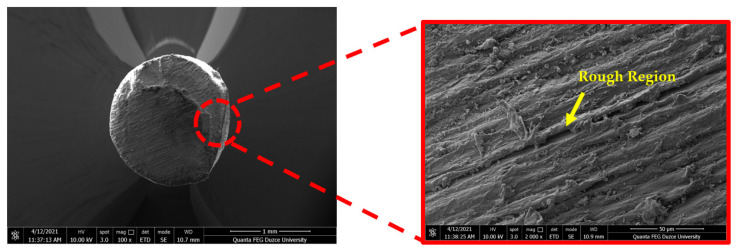
SEM micrograph of the unprinted recycled PLA (rPLA) filament prior to printing, revealing surface roughness and defects induced by the recycling process. Adopted from ref. [254].

SEM analysis of the produced rPLA filament reveals the characteristic surface roughness and minor defects induced by the recycling process prior to printing (Figure 5).

**Figure 6:**
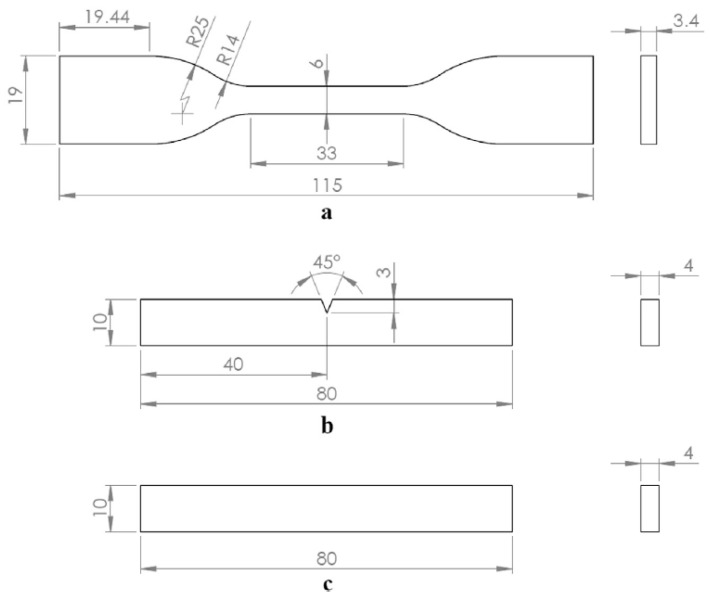
Standardized test specimen geometries and dimensions used for mechanical characterization: (**a**) tensile testing (ISO 527), (**b**) Izod impact testing (ISO 180), and (**c**) three-point bending (ISO 178). Reprinted from ref. [23].

FFF specimen fabrication: using a calibrated desktop FFF printer (Ender 3 Pro, Creality 3D, Shenzhen, China) (e.g., 0.4 mm brass nozzle, Cartesian kinematics), tensile (ISO 527 Type IV), three-point flexural (ISO 178), and Izod impact (ISO 180, Type I) specimens are printed. (Figure 6).

Parameter windows consolidated from the statistical study are as follows: nozzle 200–215 °C (PLA) or as required for composite flow; bed 60–70 °C; layer height 0.15–0.25 mm; infill 30–100% with rectilinear infill and ±45° raster orientation; print speed 40–60 mm/s; and part cooling 50–70% after first layers. Bed leveling is verified before each batch; filament diameter and ovality are checked by gauge at three points per spool. For scaffold trials, gyroid/honeycomb lattice geometries are generated to target porosity and pore size matching application constraints [189,190,191].

Characterization and analysis: Mechanical testing yields tensile strength/modulus/elongation (crosshead 5 mm/min), flexural modulus/strength (span 64 mm, 2 mm/min), and instrumented Izod impact energy. Fractography by SEM documents bead geometry, interlayer bonding and void populations, enabling correlation to process parameters. Thermal analysis combines DSC (Tg, Tm, and crystallinity) and TGA (onset temperature, residue) to quantify thermal stability and filler effects; Raman or XRD confirms crystalline phases (e.g., Al_2_O_3_, BN, and Ti-related signatures) and assesses dispersion quality. Thermal transport (for BN and graphene–alumina hybrids) is measured via laser flash or transient plane source/Hot Disk when available. Surface quality is quantified by profilometry (Ra, Rz) to capture roughness trends with recycled content and post-heat treatments.

**Figure 7:**
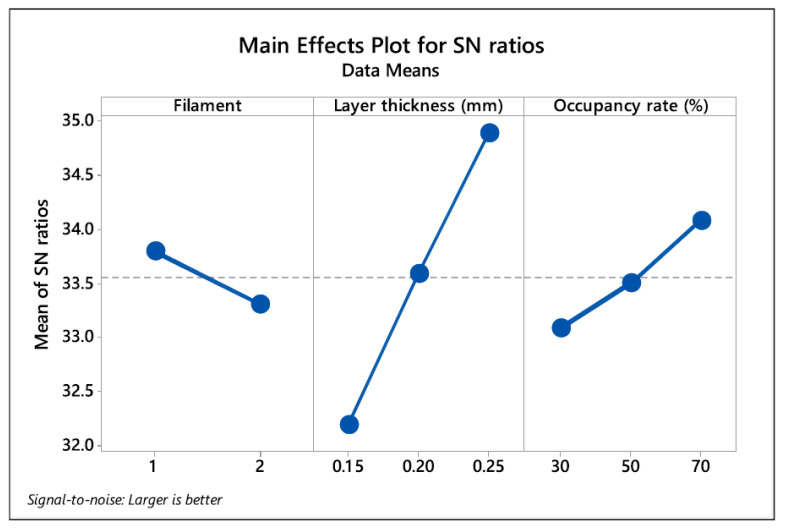
Typical Taguchi main effects plot showing the S/N ratios for mechanical strength, highlighting the relative influence of filament type, layer thickness, and occupancy rate. Adopted from [23].

Design of experiments and statistics: Factor effects are screened using Taguchi orthogonal arrays (e.g., L16/L18) with the larger-is-better signal-to-noise (S/N) metric for strength outputs. Typical factors include filament type (PLA vs. Re-PLA), layer height (0.15/0.20/0.25 mm) and infill (30/50/70%), with optional raster angle and print speed. ANOVA partitions variance: contributions consistently show layer height as the dominant effect, followed by infill density, while filament type contributes modestly under controlled moisture and diameter (Figure 7).

Linear or polynomial regressions provide predictive fits (R^2^ > 0.85 typical), and probability plots check normality of residuals. compounded in a twin-screw extruder (as detailed in Section 1.2, Figure 3).

Quality assurance and sustainability: Acceptance criteria include diameter stability and consistent flow (no stringing/clogging), dimensional tolerances within ±0.1 mm, and repeatability within ±5% for mechanical metrics. From a circularity standpoint, blends up to ~50% recycled content are prioritized to retain >90% of virgin performance under the stated parameters; when higher recycled fractions are required, increased infill and thicker layers, coupled with annealing (80–90 °C, 30–60 min), help recover interlayer bonding. Data are logged with batch identifiers to trace material history and recycling cycles.

### 2.1. Experimental Validation of the PSP Map for PLA/Re-PLA

Process tier: drying, compounding (optional reinforcements), filamentizing with diameter control and matched take-up; structure tier: bead geometry, raster overlap, void fraction governed by extrusion temperature, layer height, infill and cooling; property tier: tensile, flexural, and impact responses dominated by layer height and infill density, with recycled filaments approaching virgin performance under controlled conditions.

### 2.2. Protocol for Low-Defect Re-Extrusion and Printing

Filament production: segregation and cleaning, drying at 60–65 °C, optional twin-screw compounding with BN (1–3 wt%), Ti (1–5 wt%), Al/Al_2_O_3_ (1–4 wt%), and single-screw filamentizing with laser diameter feedback and inline filtering. Printing recipe: nozzle 200–215 °C, bed 60–70 °C, layer height 0.25 mm, infill 70%, tuned bead overlap, and optional low-temperature annealing.

From recycled PLA: near-parity mechanical properties achieved with stable diameter and pre-drying. BN-enhanced PLA for electronics: low-loading BN improves heat dissipation and dimensional stability. Ti-modified PLA for biomedical prototypes: increased ductility and potential biocompatibility at 1–5 wt%.

## 3. Results

Mechanical recycling via filament re-extrusion offers low capital requirements and localized material loops. Low-loading functional reinforcement expands reuse into thermal management, wear surfaces and biomedical prototyping. Limitations include material purity and moisture control; future work should define ultra-low filler thresholds, automate segregation, and integrate Life Cycle Assessment (LCA).

## 4. Conclusions

Evidence across recycling, re-extrusion, and FFF converges on the following four-pillar strategy: re-extrusion stability and sensing: moisture control, residence time and temperature optimization, twin-screw dispersion where needed, and in-line monitoring for filament geometry and melt characteristics [14,17,133,135,138].

Interface-aware composite design: selection of nucleating and reinforcing fillers (Al_2_O_3_, BN, Ti/TiN, and graphene) and natural fibers with appropriate surface chemistry to balance stiffness, toughness, and thermal transport without sacrificing printability [34,35,36,37,41,42,175].

Parameter optimization at the printer: controlled nozzle/bed temperatures, speeds, layer thickness, infill architecture, and build orientation, optimized with Taguchi/RSM and validated under ISO standards for tensile and impact [46,49,61,165,206,207].

Distributed recycling integration: recyclebot/FPF and maker-space workflows with energy-aware operation, quality assurance, and training, ensuring sustainable, localized manufacturing loops [89,90,212,213,214].

Within this scaffold, post-processing (annealing/heat treatment) and topology-informed design (graded infill, sandwich architectures) serve as post-print levers to consolidate performance and deliver application-ready parts even from heterogeneous recycled streams [24,44,201,226].

The literature supports the feasibility of high-value circular pathways for polymer waste through integrated mechanical recycling, filament re-extrusion, and material-extrusion AM, with PLA as a flagship platform [26,32,61,166,167,218,251,255,256].

The process–structure–property paradigm provides the conceptual foundation to navigate degradation, rheology, dispersion, and interlayer physics, while DoE/Taguchi/RSM furnish practical methods to identify robust operating windows even with recycled inputs [1,4,5,13,19,24,48,206,208,212].

Functional composites (ceramic, metallic, carbonaceous, and natural fibers) and post-processing protocols further elevate performance, expanding the application terrain from lightweight structural components to biomedical scaffolds and thermally managed devices [6,11,18,21,37,38,55,91]. Crucially, distributed recycling architectures—augmented by in-line sensing, standards-based testing, and energy-aware operation—anchor the sustainability case. Advancing this agenda will require inter-face-aware compatibilization, closed-loop process control, and hybrid mechanical/chemical upcycling to preserve or rebuild molecular weight and rheology across cycles [46,79,89,90,165,221,240]. Taken together, the reviewed evidence indicates that polymer waste can be transformed into reliable, engineered AM feedstocks, enabling circular manufacturing that is both technically rigorous and environmentally credible [31,125,168,214,222,257,258,259].

Recycled PLA can approach virgin mechanical properties when process controls and print recipes are tuned to feedstock realities. Functional reinforcement at low concentrations opens pathways to high-value reuse. The PSP framework and protocol operationalize circular polymer science transitions via recycling and reuse anchored in process engineering and standardized characterization.

Layer height (0.25 mm) and infill (70–100%) dominate tensile, flexural, and impact strength improvements across PLA/Re-PLA specimens. Low-loading reinforcements (Al_2_O_3_, BN, and Ti) provide targeted gains—rigidity/wear, thermal conduction, and biocompatible strength—without prohibitive printability losses, while graphene–alumina hybrids elevate thermal conductivity further. Blending up to ~50% recycled content retains > 90% of virgin mechanical performance when diameter stability and moisture control are maintained; higher recycled fractions show roughness and interlayer adhesion deficits mitigated by process tuning.

Strength drivers and optimized windows: Consistent with the Taguchi analysis, the layer height of 0.25 mm combined with a 70% infill delivers the highest tensile, flexural and Izod impact strengths across virgin PLA; recycled PLA follows the same trend with slightly lower absolute values. The dominance of layer height (≈70–80% contribution for tensile) reflects improved track overlap and reduced interfacial voiding at thicker layers. Infill density contributes an additional ≈10–30% depending on the property, while filament type is third-order under moisture-controlled conditions. Regression surfaces show monotonic improvements with increasing infill and layer height up to the tested bounds without over-extrusion.

Composite reinforcement effects: At low loadings, alumina nanoparticles (1–2 wt%) reinforce rigidity and wear resistance in PLA without severely penalizing ductility; percolation-like peaks are often observed around 1–2 wt% due to optimal dispersion. Titanium particulates (5–10 vol%) elevate tensile strength and compressive response and, in scaffold configurations, approach bone-like stiffness ranges while maintaining favorable cell responses reported in the literature. BN nanosheets (≈1 wt%) establish thermally conductive pathways that reduce heat accumulation during service and enhance dimensional stability; mechanical properties show a balance between stiffness gains and maintained toughness when agglomeration is avoided. Hybrid graphene–alumina systems exhibit synergistic increases in thermal conductivity compared to single-filler counterparts, provided contact resistance is minimized through network formation.

Recycling performance envelope: Blends containing 25–50% Re-PLA retain > 90% of virgin mechanical performance when filament diameter, moisture control and parameter tuning are enforced. At 100% Re-PLA, increased surface roughness and reduced strengths are attributable to molecular-weight loss and less effective interlayer bonding; mitigation strategies include annealing, higher infill and optimized cooling. SEM consistently reveals voids and interlamellar discontinuities as primary failure loci; these diminish with thicker layers and higher infill, corroborating the statistical findings.

Implications: The integrated results define a practical process–structure–property map for designing application-ready, sustainable PLA filaments. For structural parts, the 0.25 mm/70% window is a robust baseline, while composite and recycled routes allow tuning for thermal management, wear, and circularity targets.

## Figures and Tables

**Table 1:** Summary of key research studies, methodologies, and findings related to recycled polymers and circular economy strategies discussed in Section 1.

Research Focus	Key References	Methodology/Context	Key Findings and Contributions
Recycling of PLA and Circular Economy	[1,2,3,4,5,6,21]	PLA as a model biopolymer for FFF loops.	PLA is a privileged testbed due to its processability and end-of-life options.
Process–Structure–Property (PSP)	[11,13,32,33]	Analysis of chain scission and viscosity during reprocessing.	Optimized thermal control during recycling effectively mitigates property losses.
Advanced Fillers and Composites	[34,35,36,37,38]	Integration of Al_2_O_3_, BN, and Graphene into PLA matrix.	Fillers improve heat deflection and stiffness through synergistic nucleation.
Natural Fibers and Biomass	[39,40,41,42,43]	Use of flax, wood, and nutshells in recycled PLA.	Surface treatments are essential to manage moisture and improve fiber-matrix adhesion.
Post-Processing and Annealing	[24,44,45]	Thermal schedules (70–120 °C) for printed components.	Annealing reduces internal stress and restores stiffness in recycled parts.
Optimization Strategies (Design of Experiments: DoE)	[46,47,48,49]	Use of Taguchi and Response Surface Methodology (RSM).	Parameter re-centering can restore or exceed virgin PLA properties in recycled batches.

## Data Availability

The original contributions presented in this study are included in the article. Further inquiries can be directed to the corresponding author.
